# Supporting adoptive and foster parents of adolescents through the trauma-informed e-Connect parent group: a preliminary descriptive study

**DOI:** 10.3389/fpsyg.2024.1266930

**Published:** 2024-02-08

**Authors:** Cecilia Serena Pace, Stefania Muzi, Marlene Moretti, Lavinia Barone

**Affiliations:** ^1^Laboratory of Clinical Psychology (LACLIPSY), Department of Educational Sciences, University of Genoa, Genoa, Italy; ^2^Centro Italiano Aiuti all’Infanzia (CIAI), Genoa, Italy; ^3^Psychology Department, Simon Fraser University, Vancouver, BC, Canada; ^4^Laboratory of Attachment and Parenting (LAG), Department of Brain and Behavioral Sciences, University of Pavia, Pavia, Italy

**Keywords:** connect parent group, attachment-based intervention, adolescence, adoption, foster care, online intervention, parenting program

## Abstract

**Introduction:**

Adolescents in adoption and foster care are likely to show internalizing and externalizing problems and affective dysregulation, leading to a lower quality of parent–adolescent attachment relationships and high levels of strain for parents. This study describes the results of the first application of the trauma-informed attachment-based Connect Parent Group in an online form (e-Connect) with Italian adoptive and foster parents. In this study, we describe (1) trends in the aforementioned variables between pre- (T1) and post- (T2) intervention and (2) parents’ feedback and suggestions about the intervention.

**Method:**

Participants were 10 adoptive and 10 foster parents (53% females, *M*_age_ = 53.48; SD_age_ = 4.93) who attended e-Connect, an attachment-focused and trauma-informed 10-session online group intervention. This intervention aims at increasing caregiver awareness of attachment and trauma concerning adolescents’ problem behaviors and sensitive responsiveness, thereby leading to improvements in parent–adolescent relationship quality, decreases in adolescents’ problems, and reductions in caregiver strain. One e-Connect group was offered for adoptive parents and one for foster parents. Parents completed questionnaires 1 week before (T1) and after (T2) the intervention and responded to a feedback interview following program completion.

**Results:**

Only at the descriptive level, scores of adolescents’ internalizing and externalizing problems, affective dysregulation, and caregivers’ strain show decreasing trends. Parents reported high satisfaction with the program, declaring changes in parent–adolescent relationships both currently (94.7%) and anticipated in the future (100%). All parents indicated that they would recommend e-Connect to other parents.

**Discussion:**

Given promising parents’ feedback, the feasibility of e-Connect supporting adoptive and foster parents of adolescents can be further empirically investigated.

## 1 Introduction

Adolescents placed in adoption or foster care (Barroso et al., 2017; Muzi and Pace, 2023) are at high risk for childhood traumatic experiences and attachment ruptures (Pace et al., 2022) and suffer from significantly higher levels of internalizing and externalizing problems than peers raised by low-risk biological parents (Behle and Pinquart, 2016; Pace and Muzi, 2017; Pace et al., 2018; Oswald et al., 2019; Engler et al., 2022).

Moreover, both adopted and foster adolescents’ exposure to past attachment trauma can be linked to their actual attachment problems (Dozier and Rutter, 2008; Oswald et al., 2019). This may hinder them in forming and maintaining positive attachment bonds with new adult caregivers (Dozier and Rutter, 2008; West et al., 2020; Muzi and Pace, 2022), potentially increase their long-term disadvantage, and require parents’ great emotional and financial efforts. In particular, research has highlighted high levels of parental distress and caregiving strain in parents of adopted and fostered adolescents (Sánchez-Sandoval and Palacios, 2012; Goemans et al., 2020), and that adolescence is a period of increasing conflicts, parental difficulties, and emotion dysregulation in adoptive and foster families (Sánchez-Sandoval and Palacios, 2012; Golding, 2014; Leake et al., 2019).

In this regard, some studies have remarked on the importance of parental psychological support to reduce the strain on adoptive and foster parents (Golding, 2014; Oldani and Pancino, 2017; Leake et al., 2019; Hanlon et al., 2022). Reviews (Ní Chobhthaigh and Duffy, 2019; Lotty et al., 2021) highlight that tailored interventions can also be beneficial in reducing children’s emotional-behavioral difficulties, especially when they address issues of trauma and attachment, and enhance parents’ reflective engagement and skill building (Juffer et al., 2005; Barone and Ozturk, 2019; Ní Chobhthaigh and Duffy, 2019; Lotty et al., 2021; Van Ijzendoorn et al., 2023). However, from the cited reviews (Ní Chobhthaigh and Duffy, 2019; Lotty et al., 2021), none of these available interventions are delivered online, even if some studies during the COVID-19 pandemic (Oldani and Pancino, 2017; Hanlon et al., 2022) demonstrated that adoptive and foster parents particularly struggled during the pandemic due to the absence of support interventions, which had almost exclusively been provided in person to date.

Moreover, most of the available programs are not directly focused on the adolescence phase, except for the Connect Parent Group (CPG^®^) developed by Moretti and Obsuth (2009), Moretti et al. (2015), and Moretti et al. (2017) who recently implemented a trauma-informed adaptation for foster and adoptive parents (Moretti et al., 2020; Pasalich et al., 2021).

### 1.1 Support adolescent caregivers through trauma-informed and online adaptations of the connect parent group (CPG^®^)

The standard CPG^®^ is a 10-week manualized group program for parents of pre-teens and teenagers with serious behavioral and emotional problems. The program targets parenting factors that promote secure attachment, namely caregiver sensitivity, reflective functioning, shared mutuality, and dyadic affect regulation, which are crucial for supporting healthy adolescent development and autonomy while maintaining a positive emotional connection with their parents (Moretti and Obsuth, 2009; Moretti et al., 2015; Barone et al., 2020). Each weekly 90-min session is co-conducted in person by two trained group facilitators and introduces the principle of attachment. Sessions are designed to engage parents through role plays and reflection exercises that promote emotion-based learning (Barone et al., 2020). Research conducted over 15 years proved CPG^®^ as effective in building targeted parenting skills, reducing parental stress, increasing the attachment security of adolescents, and improving the quality of parent–adolescent relationships, as well as decreasing the levels of internalizing and externalizing problems in adolescents (Moretti and Obsuth, 2009; Moretti et al., 2015; Högström et al., 2017; Moretti et al., 2017; Osman et al., 2017a,b; Ozturk et al., 2019; Barone et al., 2020; Moretti et al., 2020; Pasalich et al., 2021; Pasalich and Palfrey, 2021), with positive outcomes up to 2 years after the intervention (Högström et al., 2017).

However, when the standard in-person CPG^®^ was used with foster parents, they did not find it as beneficial as biological parents. Therefore, based on foster parents’ suggestions, Moretti et al. developed a trauma-informed adaptation for foster parents of teens (Moretti et al., 2020; Pasalich et al., 2021). In this adaptation, each session integrates a part that can help caregivers understand the impact of pre-placement adverse experiences on adolescents’ behavior and deal with obstacles and feelings posed by the relationship with childcare services. For example, the first two sessions integrate psychoeducational information on how attachment can be affected by traumatic early childhood adversities, and the third session focuses on how trauma can distort adolescents’ behaviors in response to parent–adolescent conflicts. All sessions aim at enhancing the reflective engagement of the parents, considering the role of the attachment background of adolescents (their “attachment suitcase”) in shaping adolescents’ behavior, aiming at building parent skills of reflection, understanding, and sensitive response to adolescents’ behaviors. The pilot test of this trauma-informed adaptation of CPG^©^ with foster and kinship parents revealed promising results and positive feedback from parents and professionals working with them (Moretti et al., 2020; Pasalich et al., 2021), overall suggesting that this adaptation is recommended for foster and adoptive families.

Moreover, during the COVID-19 pandemic, Bao and Moretti (2023) adapted the standard intervention to be delivered online, i.e., e-Connect. In this adaptation, each participant follows the group through video calls on a personal device, a laptop, or a tablet, which enables them to see other participants and the facilitators who use digital flipcharts to work through exercises and reflections. Digital flipcharts contain prompts and are filled with participants’ responses, as would paper flipcharts in the in-person version. To prevent facilitators’ fatigue, unlike CPG^®^ in person, e-Connect is composed of smaller groups of a maximum of 10 participants and requires an additional assistant to manage the online flipcharts and provide technical support (Bao and Moretti, 2023). Role-playing has also been adapted to be performed by the two facilitators, each using their screen so parents can clearly observe facial expressions and non-verbal language. To facilitate participants’ attention and facilitators’ efficiency, for each e-Connect group, preferably no more than 10–12 video tiles should appear on the screen (Pasalich and Palfrey, 2021). Research with e-Connect conducted in Canada and Italy (Bao and Moretti, 2023; Benzi et al., 2023; Tracheggiani et al., 2023) suggests positive outcomes with this online adaptation as with the in-person one, suggesting its use is promising when parents can have difficulty accessing in-person programs. For instance, the online intervention could be fruitful for adoptive and foster families, often residing in decentralized areas, while adoption/foster services are usually in urban centers (Oldani and Pancino, 2017) and the overload of commitments partly resulting from adolescents’ problems can be a barrier to accessing support services (Golding, 2014; Leake et al., 2019).

To date, the trauma-informed adaptation of the CPG^®^ has been provided only in person and only to foster parents, and there is no information about how this adaptation might work if it were also delivered online, not only to foster parents but also to adoptive ones.

### 1.2 The current study

From the above, the utility of parent support tailored to the specific needs of adoptive and foster families emerged, particularly in trauma-informed attachment-based group programs such as CPG^®^. The lack and potential of online intervention among these populations of parents also emerge. Therefore, the current study aimed to describe the outcomes of two pilot experiences where the trauma-informed adaptation of CPG^®^ for foster parents was delivered online, i.e., e-Connect for foster parents, to two groups of adoptive and foster parents of adolescents living in Italy. With descriptive purpose, we visually inspected trends of adolescents’ emotional-behavioral problems and affective regulation, caregivers’ strain, and quality of parent–adolescent relationships from the week before the intervention (T1) to the week after the intervention (T2), and we reported their feedback and suggestions about the program’s value, strengths, and limits.

## 2 Materials and methods

### 2.1 Participants and procedure

The study was conducted with the support of “Fondo di Beneficenza di Intesa Sanpaolo” (protocol n. B/2021/0452).

**Figure 1:**
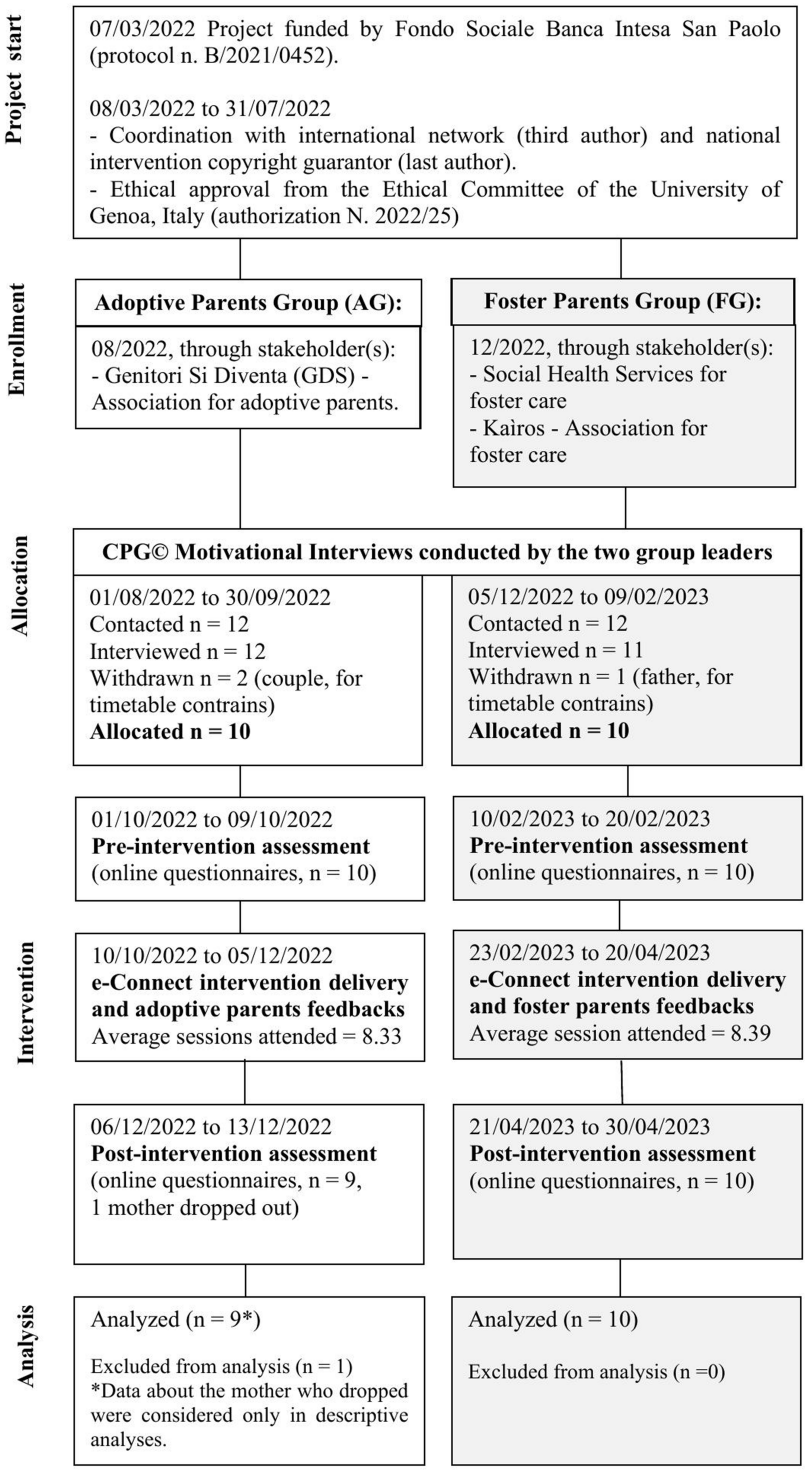
Flowchart of the study procedure.

Participants were enrolled with the collaboration of the project stakeholders, who signed a formal agreement sheet to collaborate. Full details about the study procedure (time and phases of data collection, dropout and attrition, and data collected and analyzed) are shown in the flowchart in Figure 1.

Participants reported on socio-demographic information during pre-intervention interviews, which are included in the Connect program and are designed to increase treatment motivation and address barriers to attendance. Socio-demographic information was also collected in the set of T1 pre-intervention questionnaires. This study included data from parents who completed both pre- and post-intervention questionnaires and at least 70% of sessions, which were all participants except one adoptive mother, who dropped at the second session for personal unresolvable reasons (5%). Therefore, this study included data of 9 adoptive parents (5 fathers, *M*_age_ = 51.30, SD_age_ = 5.75) of 6 adopted adolescents (60% males, *M*_age_ = 13.60, SD_age_ = 1.67; placed at *M*_age_ = 6.42 for *M*_lenght_ = 7.40; area of origin: 60% Italy, 40% Russia, 20% Colombia), and of 10 foster parents (four fathers, *M*_age_ = 55.30, SD_age_ = 4.29) of 8 adolescents in foster care (50% males, *M*_age_ = 14.13, SD_age_ = 3.04; placed at *M*_age_ = 6.42 for *M*_lenght_ = 7.88; area of origin: 87.5% Italy, 12.5% Pakistan). All parents had medium-high socio-educational levels. Only foster parents already had biological siblings (70%).

Data from the two samples were reported separately to highlight potential population specificities and because of different population characteristics, i.e., legal parenting in adoption vs. a temporary role in foster care.

### 2.2 Measures

#### 2.2.1 Adolescents’ problems and affect regulation referred by parents

The 25-item Strengths and Difficulties Questionnaire filled out by parents (SDQ-parent version) (Goodman et al., 2010) was used to assess adolescents’ symptoms as evaluated by parents. It provides two scores for internalizing problems (sum of emotional problems and peer problems subscales) and externalizing ones (sum of conduct problems and hyperactivity-inattention subscales).

The 12-item Affect Regulation Checklist Youth (ARC-Y) (Moretti et al., 2015; Goulter et al., 2023) was completed by parents to assess adolescents’ affect regulation in the previous 6 months in the dimensions of affect dysregulation, reflective strategies, and affect suppression.

#### 2.2.2 Parental self-reported strain and opinion of parent–adolescent relationship quality

The Caregiver Strain Questionnaire-Short Form (CGSQ-SF) (Brannan et al., 1997), a 10-item self-report questionnaire on a 5-point Likert-type scale, was used. The measure assesses strain on three dimensions: caregivers’ subjective internalized strain, subjective externalized strain, and objective strain, and provides a score in each of these three dimensions as the mean of the scale items’ scores, plus a global score of strain as the sum of scores in the three dimensions.

The self-report questionnaire Psychological Availability and Reliance On Adult–Parent version (PARA-P) (Schuengel and Zegers, 2003; Muzi and Pace, 2020), an 18-item questionnaire on a 4-point Likert scale where the caregiver assesses the quality of the adolescent–adult relationship in three scores of psychological availability, reliance on adults, and affectional bond, corresponding to the mean of the item scores in each dimension. The mean of scores in the first two dimensions provides an additional score of attachment security. In the PARA-P, item 8 of the original 19-item version must not be administered.

#### 2.2.3 E-connect feedback and integration

Parents’ evaluation of e-Connect was collected during the 10th session of “Feedback and Integration,” where parents completed a 15-item questionnaire plus a group semi-structured interview conducted by an independent research team member about the perceived value of intervention to understand and respond to their adolescents’ problems and suggestions to improve the intervention.

### 2.3 Analytic plan

This study includes data from T1 and T2, as the follow-up data collection (T3) is ongoing. Given the descriptive nature of the study and the limited sample sizes, no statistical analyses were performed. Scores in the two phases in each group were reported in a table, and results of the feedback session were narratively reported, grouped in themes of “program value,” “perceived changes,” and “suggestions to improve.”

## 3 Results

### 3.1 Trends from pre- (T1) to post- (T2) intervention

Table 1 reports scores’ means [*M*] and standard deviations [SD] in both groups.

**Table 1:** Scores at pre-intervention (T1) and post-intervention (T2) of parents attending the trauma-informed adaptation of e-Connect for adoptive and foster parents.

	Adoptive parents group				Foster parents group			
	T1		T2		T1		T2	
	*M*	*SD*	*M*	*SD*	*M*	*SD*	*M*	*SD*
Adolescent symptoms (SDQ)								
Internalizing problems	6.90	3.90	6.22	4.79	5.10	2.13	4.30	2.87
Externalizing problems	12.30	3.86	10.78	4.55	9.60	3.69	8.20	3.26
Adolescent affective regulation (ARC-Y)								
Affective dysregulation	13.20	4.16	11.00	3.67	12.70	3.92	12.40	3.44
Reflective strategies	11.90	2.42	10.78	2.39	13.00	3.65	12.10	2.23
Affective suppression	10.00	3.30	9.67	3.67	11.50	3.41	11.20	3.05
Caregiver strain (CSQ)								
Objective strain	2.38	0.87	2.06	0.87	1.59	0.47	1.56	1.59
Subjective internalized strain	2.48	0.38	2.72	0.42	2.75	0.44	1.90	2.75
Subjective externalized strain	2.65	0.91	2.20	0.88	2.05	0.57	2.08	2.05
Caregiver strain total	7.51	1.16	6.99	1.91	6.39	0.91	5.55	6.39
Quality of parent–adolescent relationship (PARA)								
Psychological availability	3.44	0.47	3.39	0.42	3.08	0.48	2.98	0.41
Reliance on adult	2.51	0.24	2.59	0.29	2.53	0.22	2.77	0.46
Security	2.98	0.27	2.99	0.28	2.80	0.29	2.88	0.39
Affectional bond	2.86	0.30	2.91	0.40	2.64	0.34	2.73	0.40

Adoptive parents group *n* = 9, foster parents group *n* = 10.

Scores on Table 1 seem to suggest decreasing trends regarding scores of parent-reported adolescents’ internalizing and externalizing problems and affective dysregulation and suppression, as well as in caregiving strain, while lines of quality parent–adolescent relationships appear quite stable on high scores (over 2.5 on a 4-point Likert scale).

### 3.2 Parents’ feedback

#### 3.2.1 Program value

Most parents reported that e-Connect was quite or very useful (89.4%) in understanding their child, and similarly, they reported that it was quite or very useful in understanding themselves (84.2%). Most parents reported as strengths the chance to “normalize” and “understand” problem behaviors of children that often lead to confusion and a sense of powerlessness, e.g.*, [it was useful to]“understand and accept his aggressivity as a way to manifest his needs and not a violence toward me”* or “*recognize that every behaviour has an origin and our children are not foolish*,” as well as the attachment perspective in discussing certain topics, e.g., “*the value and the sensitive management of the conflict and periods of impasse*” or “*understand that relationship’s crisis can be a resource*” and “*it has reassured me that there are not only his needs but also mine*”.

All parents (100%) reported the discussion of trauma effects and attachment, empathy, and conflict, as well as role-playing, as the most useful dimensions of the intervention. All of them would suggest other adoptive and foster parents attend e-Connect (100%).

#### 3.2.2 Perceived changes

Most parents (94.7%) reported changes in their parent–adolescent relationship as an effect of having attended e-Connect, e.g., “*In the past, I would have overreacted in front of poor transparency of [my son], I would have taken it personally, but now I have understood that he avoids conflicts because in his history this would have mean be abandoned or beaten […] he said me ‘mom, you are a better mom since you attend that group!’*” or “*I sought the magic and…I found it! [During the group] I have started to reduce the volume of the radio when he seems to want to talk, and our relationship is much better now!*” or “*I am more cautious and I put more effort in listening to my daughter’s opinion before jumping to the conclusions*.” All of them forecasted further changes in the future (100, 57.9% very much, and 42.1% quite much). Most of them referred to feeling more confident in their parental abilities after the intervention (68%), e.g.*, “I was already prepared about [my son] bruises/traumas, but I improved the way of managing it*.*,”* or *“I increased the observation and curiosity of my child.”*

#### 3.2.3 Suggestions to improve

Almost all parents suggested proposing the intervention before the placement and/or during early adolescence. Some parents suggested enlarging the focus to current problems such as the adolescents’ misuse of social networks or external relationships with peers and teachers, and some of them reported a supposed preference for in-person delivery.

## 4 Discussion

This study aimed to describe the first employment of trauma-informed and attachment-based parenting interventions for foster parents of teens (Moretti and Obsuth, 2009; Moretti et al., 2015, 2017) in an online form, i.e., e-Connect for foster parents. This adaptation was proposed to two small groups of adoptive and foster parents in Italy, who provided feedback and suggestions about the intervention’s value, strengths, and limits. This study has a descriptive goal, describing trends in youth and parent difficulties and parent–adolescent relationships from the week before and after the intervention and reporting parents’ feedback about the intervention. Therefore, the following comments are qualitative, with no statistical relevance, but with the idea of understanding if this intervention may deserve further empirically based investigation and feasibility testing.

Apparently, scores in Table 1 suggest a reduction in all the outcomes inquired, i.e., adolescents’ internalizing and externalizing problems, affective dysregulation, and caregiving strain. Future studies with larger samples are required to confirm or disconfirm these results on a statistical basis. In this regard, the scores of adoptive and foster parents presented some difference, and an empirical investigation should clarify if the intervention worked differently in the two samples, highlighting some population-specificity not detected in the feedback session, where both groups reported similar contents.

Concerning comments provided by both adoptive and foster parents during the feedback session, they were mostly positive and overall reflected a high level of satisfaction with having participated in e-Connect for foster parents. In fact, in the feedback session, both adoptive and foster parents mainly highlighted that e-Connect helped them to improve their ability to “stop” and “get curious” when faced with their children’s problematic behaviors, in terms of aggressive and oppositional-defiant behaviors as well as lying and lazy withdrawal, instead of “jumping to conclusions” and reacting angrily or confused to these problematic behaviors. Furthermore, they emphasized that receiving psychoeducation on the lasting effects of early adverse experiences on behavior and attachment improved their empathy toward their children’s harsh history and relieved them of guilt about adolescent problems or anger toward children, clarifying the involuntary nature of their problematic behaviors and relative independence from the attitudes of adoptive and foster parents. Thus, in the feedback session, parents mostly reported positive changes in the relationship and the expectation of changes in the future. The contents of this feedback align with those of foster parents attending the in-person version of the intervention (Moretti et al., 2020; Pasalich et al., 2021) and, overall, suggest a perceived value of e-Connect for foster parents in supporting adoptive and foster parents during adolescence. Our study calls for further empirical investigation on the feasibility of the eConnect online version compared to the traditional CPG^®^ in-person with these populations, paying particular attention to its strengths and limitations. An obvious strength of this treatment format is the potential to reach decentralized families who can participate in treatment from their own homes. This is often a better fit for many parents, and many parents voiced this to us informally. On the other hand, some parents highlighted that physical distance slightly limited their motivation to engage in role-playing as an actor with the co-leader, creating a slight emotional barrier, especially in the first sessions, which may have slowed the development of group cohesion. Over time, however, we found that parents became increasingly comfortable participating by sharing their perspectives and experiences with other parents. In this regard, a central factor may have been the positive attitude of the co-leaders toward the delivery of the online intervention, as the comfort of the therapist is proven crucial in favoring clients’ engagement in the online intervention (Cowan et al., 2019), calling for future training on how to deliver psychological interventions online.

### 4.1 Limitations and conclusion

This descriptive study has marked limitations to consider. First, because of the absence of data from follow-up assessment (under collection), this study was designed as a pilot preliminary description, and no statistical analyses were performed, so the statistical significance of the trends described was not empirically tested and should be verified by future studies. With the scope to describe pilot experiences and highlight possible population specificities, the small sample size groups were kept separate because they participated in the intervention separately and because of population characteristics, i.e., legal parenting in adoption vs. a temporary role in foster care. Furthermore, some parents were couples with only one child and referred to the same children, so the data were not independent of each other, and a larger, statistically based investigation should address this point. Third, adoptive and foster families have been fairly supported from the beginning, benefiting from psychological support and declaring a good-quality parent–adolescent relationship since T1. Therefore, this sample may not totally represent hard-to-reach adoptive and foster parents dealing with the plight of their children included in the previously mentioned CPG^®^ and e-Connect studies. Some families may be hard to reach due to their demanding childcare responsibilities that often occur as a result of problems in parenting adopted children (Moretti and Peled, 2004; Sánchez-Sandoval and Palacios, 2012; Golding, 2014; Oldani and Pancino, 2017; Leake et al., 2019; Hanlon et al., 2022) or because of their distant or remote geographical location. This online intervention is a promising option for overcoming these population-specific barriers to treatment. Furthermore, by working in partnership with formal and informal agencies and local services, we have found that barriers to the recruitment of population-specific and hard-to-reach populations (van der Ven et al., 2022) can be more easily resolved. Importantly, this recruitment strategy can build the engagement of trusted community professionals who can encourage and support parents in enrolling in online treatment (van der Ven et al., 2022).

However, having only one group of adoptive parents and one of foster parents, this study is configured more as the description of a pilot experience, and further experiences of other groups in these populations are necessary in order to reach the numbers necessary to substantiate these results with appropriate statistical tests.

Finally, because the aim was not a statistical pre- and post-change investigation, we did not report information from adolescent measurements or data from a control group, all limitations to be addressed in future study design.

Despite these limitations, and given the published findings supporting the effectiveness of Connect for alternate caregivers (Pasalich and Palfrey, 2021) and e-Connect in other countries (Bao and Moretti, 2023), our results appear encouraging and suggest further research on the feasibility of the trauma-informed version of e-Connect with adoptive and foster parents in Italy.

## Data availability statement

The raw data supporting the conclusions of this article will be made available by the authors, without undue reservation.

## Ethics statement

The studies involving humans were approved by Università degli Studi di Genova Comitato Etico per la Ricerca di Ateneo (CERA). The studies were conducted in accordance with the local legislation and institutional requirements. The participants provided their written informed consent to participate in this study. Written informed consent was obtained from the individual(s) for the publication of any potentially identifiable images or data included in this article.

## Author contributions

CP: Conceptualization, Data curation, Funding acquisition, Investigation, Methodology, Project administration, Resources, Supervision, Validation, Visualization, Writing – original draft, Writing – review & editing. SM: Data curation, Formal analysis, Investigation, Methodology, Resources, Software, Validation, Visualization, Writing – original draft, Writing – review & editing. MM: Conceptualization, Methodology, Visualization, Writing – review & editing, Supervision. LB: Conceptualization, Methodology, Resources, Supervision, Visualization, Writing – review & editing.
